# FAM21C Promotes Hepatocellular Carcinoma Invasion and Metastasis by Driving Actin Cytoskeleton Remodeling *via* Inhibiting Capping Ability of CAPZA1

**DOI:** 10.3389/fonc.2021.809195

**Published:** 2022-01-13

**Authors:** Yao Lu, Deng Huang, Baolin Wang, Bowen Zheng, Jialong Liu, Juxian Song, Shuguo Zheng

**Affiliations:** ^1^ Institute of Hepatobiliary Surgery, Southwest Hospital, Third Military Medical University (Army Medical University), Chongqing, China; ^2^ Department of Hepatobiliary, General Hospital of Tibet Military Command Area, Tibet, China

**Keywords:** hepatocellular carcinoma, FAM21C, CAPZA1, actin cytoskeleton, invasion and metastasis

## Abstract

Hepatocellular carcinoma (HCC) is characterized by a high incidence of metastasis. The dynamic remodeling of the actin cytoskeleton plays an important role in the invasion and migration of HCC cells. In previous studies, we found that CAPZA1, a capping protein, can promote EMT of HCC cells by regulating the remodeling of the actin filament (F-actin) cytoskeleton, thus promoting the invasion and migration of HCC cells. In this study, we found that FAM21C may have a regulatory effect on CAPZA1, and we conducted an in-depth study on its potential regulatory mechanism. First, we found that FAM21C is highly expressed in HCC tissues and its high expression could promote the malignant progression of HCC. Meanwhile, the high expression of FAM21C promoted the invasion and migration of HCC cells *in vitro* and *in vivo*. Further, FAM21C interacted with CAPZA1, and their binding inhibited the capping capacity of CAPZA1, thus promoting the invasion and migration of HCC cells. This effect of FAM21C was abolished by mutating the CP-interacting (CPI) domain, the CAPZA1 binding site on FAM21C. In conclusion, high expression of FAM21C in HCC tissues can promote malignant progression of HCC and its potential mechanism involves FAM21C inhibition of CAPZA1 capping capacity by binding to CAPZA1, which drives F-actin cytoskeleton remodeling, and thus promotes invasion and migration of HCC cells.

## Introduction

HCC is the fifth most common tumor in the world and the second leading cause of cancer-related deaths (1), and its high malignancy poses a serious threat to human health. Due to hepatitis B virus infection and aflatoxin exposure, China has become a region with the highest incidence of HCC (2). Currently, radical resection is still the most effective treatment for HCC, but its postoperative survival rate at five years is only about 25% to 50%. The appearance of invasion- and metastasis-related phenotype, such as multifocal sites and invasion of the main vessel in HCC predicts the poor prognosis of patients (3). Therefore, it is very important to investigate the molecular mechanisms of invasion and migration of HCC cells to identify the corresponding therapeutic targets with the objective of improving the prognosis of patients.

HCC invasion and metastasis is a complex biological behavior of cells that involves multiple signaling pathways (4). The actin cytoskeleton not only serves as a reticular scaffold supporting cell space, but also participates in the regulation of a variety of cell biological behaviors, including migration, invasion, and cargo transport (5–7). Studies have shown that dynamic remodeling of the cytoskeleton plays an important role in the invasion and migration of tumor cells and is becoming a major focus of current cancer research. It has been reported that actin cytoskeleton remodeling promotes HCC invasion and metastasis by participating in biological events such as epithelial-mesenchymal transition (EMT), invadopodia formation, and endocytic recycling of specific cargo in HCC cells (8–10). These findings suggest that an in-depth study of the cytoskeletal remodeling mechanism during tumor cell invasion and migration may provide new ideas to reveal the mechanism of tumor invasion and metastasis. Our previous study showed that the α1 subunit of the cytoskeletal protein CAPZ (CAPZA1), which can directly bind to F-actin, has low expression in HCC tissues and can also participate in the regulation of actin filament cytoskeleton remodeling to promote EMT in HCC cells (11). Therefore, we suggest that the mechanism of CAPZA1 involves the inhibition of further lengthening of F-actin by binding to the barbed end of actin filaments, which induces cytoskeleton remodeling and thus inhibits invasion and migration of HCC cells. However, it has not been reported whether CAPZA1 is regulated by other upstream molecules in HCC.

FAM21C, also known as vaccinia virus penetration factor (VPEF), because it helps the vaccinia virus enter HeLa cells by liquid-phase endocytosis (12). Subsequently, it was reported that the WASH (WASP and SCAR homologue) complex is an important member of the WASP family, consisting of five subunits including FAM21 and containing the VCA domain, which can achieve regulation of site-specific actin polymerization by recruiting and activating the Arp2/3 complex; FAM21C is a subunits of the WASH complex and plays an important role in maintaining the ability of the WASH complex to promote the localized F-actin polymerization (13, 14). Currently, the role of FAM21 in tumors is unclear. Studies have confirmed that the knockdown of FAM21 inhibits the migration of prostate cancer cells, and its expression is regulated by the nuclear translocation of IGFR (15); in pancreatic cancer studies, nuclear FAM21 was found to regulate NF-κB transcription, and its reduced expression increased the sensitivity of pancreatic cancer cells to gemcitabine and pentafluorouracil (16); knockdown of FAM21 expression in breast cancer cells significantly reduced the ability of cells to degrade the extracellular matrix (17). These findings suggest that FAM21C may be an important regulator in promoting tumor cell invasion and migration, but its underlying molecular mechanisms have not been clearly reported. It is reported that Fam21-tail could interact with CAPZa in HeLa cells (18). Moreover, through the STRING database (19), we found that FAM21C could interact with CAPZA1, and it is unclear whether this interaction promotes the invasion and migration of HCC cells. Therefore, this study aimed to investigate the biological functions of FAM21C in HCC and the potential molecular mechanism involved in regulating the remodeling of the actin cytoskeleton induced by CAPZA1 to promote the invasion and migration of HCC cells.

## Materials and Methods

### Bioinformatics Analysis

The Cancer Genome Atlas (TCGA) visualization tool found on the GEPIA (http://gepia.cancer-pku.cn/index.html) website was used to analyze the differences in mRNA expression levels between 369 HCC tissues and 50 normal liver tissues of FAM21C, and the realtionship between FAM21C mRNA and ACTB, as well as the relationship between mRNA levels and tumor stage and the overall survival (OS) rate in HCC. This is done by enter the FAM21C in the “Search” field, and the analyses was performed *via* different option. Then the statistical graphs were generated directly. The differences in FAM21C protein levels in HCC and liver tissues were analyzed using the Human Protein Atlas (HPA) website (https://www.proteinatlas.org/). OS and disease-free survival (DFS) associated with FAM21C in HCC were analyzed by Kaplan–Meier Plotter (http://kmplot.com/analysis/) website with the option “Automatically select the best cut-off value”. The Ualcan online bioinformatics website (http://ualcan.path.uab.edu/analysis.html) used the same approach to obtain results of FAM21C on OS from the LIHC database. The FAM21C protein interaction network was analyzed by STRING (https://string-db.org/) and GeneMANIA (http://genemania.org/) website in the same way as above.

### Cases and Follow-Up

In this study, we collected pathological specimens from 129 patients who had undergone hepatectomy for HCC at the Southwest Hospital (Chongqing, China) from January 2010 to December 2012. The patients were followed for 5 years, and clinicopathological data including age, sex, tumor size, TNM stage, tumor classification, lymphatic metastasis, Vascular invasion, intrahepatic metastasis, postoperative recurrence, postoperative survival time were collected through medical record systems and follow-up. This study was approved by the Institutional Research Ethics Committee of Southwest Hospital (KY2020127).

### Immunohistochemical Staining Analysis

We collected 129 specimens from HCC patients as paraffin tissue sections, which were then used in tissue microarrays (TMA). After dewaxing and hydration, the chips were microwave heated in sodium citrate solution to repair the antigen. Subsequently, endogenous peroxidase activity was removed with 3% hydrogen peroxide at room temperature for 30 min, while 10% BSA was used to block tissue at room temperature for 1 h. TMAs were incubated with anti-FAM21C antibody (1:500, Biorbyt, UK) at 4°C overnight. The next day, the immunohistochemical staining kit (Proteintech, China) was used for DAB staining according to the kit instructions. After dehydration, the slices were sealed with neutral resin. Each tissue was scored by 2 independent pathologists according to the following methods: tissue staining intensity scoring: 1 (+); 2 (++); 3 (+++) and positive cell ratio scoring: 1 (0-25%); 2 (26%-50%); 3 (51%-75%); 4 (>75%). The immunohistochemistry score is the product of the staining intensity score and the positive cell ratio score.

### Cell Line

Huh7 was obtained from the Fudan Cell Bank (China, Shanghai) and HepG2 was obtained from the American Type Culture Collection (ATCC). All cells were free of mycoplasma contamination. Both cell cultures were cultured with Dulbecco’s modified Eagle’s medium (DMEM) (Gibco) containing 10% fetal bovine serum (FBS) (Gibco). All cells were maintained at 37°C, 5% CO_2_ in an incubator.

### Lentivirus Infection

The FAM21C protein sequence was taken from the UniProt database (NM_015262). FAM21C shRNA, overexpression and mutation recombinant lentivirus (sh-FAM21C, FAM21C-OE, FAM21C△) was constructed, packaged, amplified by Shanghai Genechem Co Ltd. Scramble shRNA (sh-NC, LV-NC, NC-FAM21C△) served as a negative control. FAM21C overexpression and mutation lentiviruses expressed a fusion-HA/Flag-tagged protein, respectively. Specifically, amino acids were mutated at positions 1003, 1010, and 1019 to alanine (**Supplementary Figure 1**). Huh7 and HepG2 cells were seeded in 6-well plates and lentivirus transfection was performed when cells reached 20% to 30% confluence. Cells were replaced with 1 mL of fresh complete medium and the corresponding volume of lentivirus and transfection reagent was added to each well according to the instructions. Cells were selected with puromycin. The transfection efficiency was observed by fluorescence microscopy, followed by western blotting experiments to detect the expression of FAM21C and tagged proteins.

### Transwell and Invasion Assay

Transwell chambers (8.0 μm, Corning Life Science, USA) were inserted in 24-well plates (Nest, China). A 200 μL volume of serum-free medium containing 1×10^5^ cells and 600 μL of complete medium containing 10% FBS were added to the upper and lower chambers of each well, respectively. The cells were further incubated for 24 h. The cells were fixed in 4% paraformaldehyde at room temperature for 30 min, followed by crystalline violet staining (Beyotime, China) for 30 min. The cells in the upper chamber were then washed in PBS, and were gently wiped with a cotton swab. Three fields of view were randomly selected under the microscope to observe the cells and were photographed for counting (20x magnification). For invasion experiments, Matrigel (Corning Life Science, USA) was mixed with DMEM in a ratio of 1:6 and 30 μL was added to each chamber and placed in an incubator at 37°C for 5 h. The remaining steps were performed as for Transwell experiments.

### Wound Healing

Huh7 and HepG2 were inoculated in 6-well plates and cultured until the cells reached 100% confluence. Using a pipette tip, the surface of the cell monolayer was scratched in a straight line. After washing the cell debris with PBS, the culture was changed to serum-free medium and continued for an additional 24 h. The migration area of each group was observed under the microscope and was photographed (20x magnification). Microscopic images of Huh7 and HepG2 were collected at 0, 24, and 0, 30h, respectively.

### Extraction of Cytoplasmic and Cytoskeletal Proteins

The cytoplasmic and cytoskeletal proteins were extracted with the Subcellular Structure Protein Extraction Kit (Sangon, China) according to the manufacturer’s instructions. A standard number of cells (2×10^6^) were used in each sample. Each sample was mixed with 500μL of cold Extraction buffer 1 supplemented with 5μL of protease inhibitor and shaken on ice for 10min. The supernatant was collected and saved after centrifugation at 3000 rpm for 8min at 4°C. The cytoplasmic proteins were present in the supernatant. The residual precipitation was re-suspended with 200 μL of Extraction buffer 4. Then, the sample was centrifuged at 12000 g for 15 min at 4°C. The residual precipitation was dissolved with 200μL of 1×loading buffer after washing twice with −20°C with 90% acetone. The cytoskeletal proteins were dissolved in loading buffer. The protein levels were detected by western blotting (20).

### Western Blotting

Huh7 and HepG2 cells were lysed with RIPA buffer (Beyotime, China) containing protease inhibitors or phosphatase inhibitors (Beyotime, China) for 30 min on ice. The cell lysate was centrifuged at 13000 ×g for 15 min at 4°C, and the supernatant was collected. F-actin (cytoskeletal proteins) were extracted followed by *Extraction of cytoplasmic and cytoskeletal proteins*. The lysate was heated at 100°C in a metal bath for 5 mins after mixing with 5× loading buffer. Proteins were separated by SDS-PAGE and then transferred to PVDF membranes (Millpore, USA). A solution of 5% skimmed milk was used to block the membranes at room temperature for 1 h and then incubated with a primary antibody at 4°C overnight. The next day, after washing the membranes three times with TBST, the PVDF membranes were incubated with homologous HRP-conjugated secondary antibody at room temperature for 1 h. Finally, the blots were visualized with ECL reagent using an imaging system (Vilber, France). Antibody descriptions were as follows: (FAM21C:1:1000, Millpore, USA; GAPDH:1:10,000, Proteintech, China; HA:1:1000, Roche, Switzerland; F-actin:1:1000, Abcam, USA; CAPZA1:1:5000, Abcam, USA; Flag:1: 1000, Sigma, USA).

### Co-Immunoprecipitation

The Protein A/G magnetic beads were obtained from Biomake. A 50 μL volume of beads were transferred to eppendorf tubes, after three washes in TBST, 200 μL PBS containing 10 μL of anti-CAPZA1 antibody (Abcam, USA) or anti-HA antibody (Roche, Switzerland) was added to eppendorf tubes, and shaken at room temperature for 1 h. After washing with TBST three times, the beads were resuspended in 500 μL of the antigen-containing lysate, and shaken at 4°C overnight. The next day, the supernatant was discarded by magnetic separation, TBST washed three times and then 35 μL of 1×loading buffer was added and heated at 100°C in a metal bath for 10 min. The supernatant was collected in another eppendorf tube. The remaining steps were the same as in those for western blotting. For quantitative Co-immunoprecipitation, an equal number of cells with different treatments were extracted. Protein concentrations were determined using the BCA Protein Assay Kit (Beyotime, China). CAPZA1 was considered as a loading control. The remaining steps were the same as for Co-IP.

### Immunocytofluorescence

Huh7 cells were seeded in 24-well plates containing clean coverslips. After washing with PBS 3 times, cells were fixed in 4% paraformaldehyde and permeabilized with 0.2% Triton-100, then blocked with 5% BSA for 1 h at room temperature. Subsequently, the coverslips were incubated with primary antibody (FAM21C: 1:50, Biorbyt, UK) at 4°C overnight. The next day, the primary antibody was discarded and the cells were washed 3 times with PBS and incubated with Alexa Fluor 488-conjugated secondary antibodies (1:200, Proteintech, China) in a wet box for 1 h. Subsequently, cells were stained with TRITC Phalloidin (1:200, Solarbio, China) for 30 min at room temperature to detect the actin cytoskeleton. Finally, 10 μL of antifluorescence quencher containing DAPI was used to stain the nuclei. Cells were examined by fluorescence microscopy and photographed (40x magnification).

### Orthotopic Xenograft Model

SPF-grade BALB/c nude mice were used to establish the orthotopic xenograft model(6-week old, males, each group n=6). Nude mice were anesthetized with isoflurane by inhalation, a 1-cm incision was made in the midline of the abdomen to expose the left lobe of the liver, and sh-FAM21C-expressing Huh7 and negative control cells (1×10^6^ cells/80 μL DMEM, containing 30 μL of Matrigel) were injected with a microinjector under the liver envelope. Continuous sutures closed the abdomen. A normal diet was maintained and mice were observed every 2 days. Six weeks later, the nude mice were euthanized under deep anesthesia, the livers were removed and photographed, and the number of metastases was counted, after which tissues were preserved in 4% paraformaldehyde. then, the liver was sectioned and stained with haematoxylin and eosin, and tumor lesions were observed. Animal experiments were approved by the Laboratory Animal Welfare and Ethics Committee of the Third Military Medical University (Army Medical University), chongqing, China.

### Statistical Analysis

All statistical analyses were performed with GraphPad Prism 6.0 (GraphPad Software Ltd, San Diego, CA). Images were processed with ImageJ free software. The data were expressed as mean ± standard deviation. Comparisons between two groups were evaluated using independent sample *t* tests or paired-sample *t* tests. The Chi-square test was used to analyze the relationship between FAM21C and clinicopathological parameters. Survival analysis was performed using Kaplan–Meier survival analysis. *P*–value less than 0.05 (*P*<0.05) was considered significant.

## Results

### Bioinformatics Analysis Suggested That FAM21C Expression Was Closely Related to the Malignant Progression of HCC

**Figure 1:**
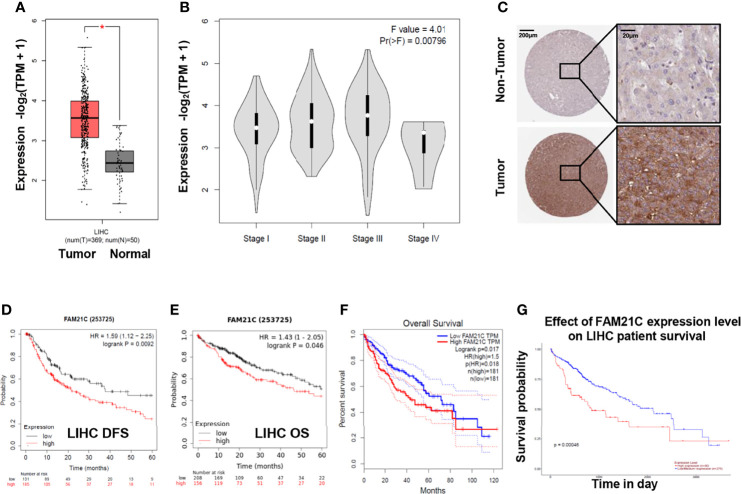
FAM21C increases in HCC and is an indicator a poor prognosis by bioinformatics analysis. **(A)** The differential analysis of mRNA levels of FAM21C between 369 HCC tissue and 50 normal tissues. **(B)** Relationship between FAM21C mRNA expression levels and the HCC clinical stages in GEPIA datasets. **(C)** The protein level of FAM21C in HCC tissue and non-tumor tissues using the Human Protein Atlas (HPA) database. Representative pictures are shown. **(D, E)** DFS and OS of FAM21C in Kaplan–Meier Plotter database using the “Auto select best cutoff” option. **(F, G)** The OS of FAM21C in GEPIA and Ualcan database respectively. Survival analysis showed that high expression of FAM21C mRNA indicated a poor survival time. *P < 0.05 was considered statistically significant.

To gain a preliminary understanding of the role of FAM21C in HCC, we analyzed 369 HCC tissues and 50 normal liver tissues from the online database GEPIA (21), and FAM21C mRNA was found to be highly expressed in HCC tissues (**Figure 1A**). Meanwhile, the expression of FAM21C mRNA gradually increased with increasing TNM stage (I–III) of HCC but the elevated expression in stage IV HCC tissues was not significant (*P*=0.00796) (**Figure 1B**). Furthermore, we searched for the expression of the FAM21C protein in HCC tissues and normal liver tissues in the HPA database and found that the immunohistochemical staining signal of the FAM21C protein was stronger in HCC tissues (representative images are shown) (**Figure 1C**) (22). Finally, we analyzed the effect of FAM21C on the prognosis of HCC patients from the GEPIA, Kaplan–Meier Plotter, and Ualcan databases, and the results showed that the OS and DFS were significantly lower in the FAM21C mRNA high-expression group than in the FAM21C mRNA low-expression group (**Figures 1D–G**) (23). The results of the above bioinformatics analysis suggested that the high expression of FAM21C in HCC could be closely related to the malignant progression of HCC.

### Case Analysis Confirms That High Expression of FAM21C in HCC Tissue Promotes Malignant Progression of HCC

**Figure 2:**
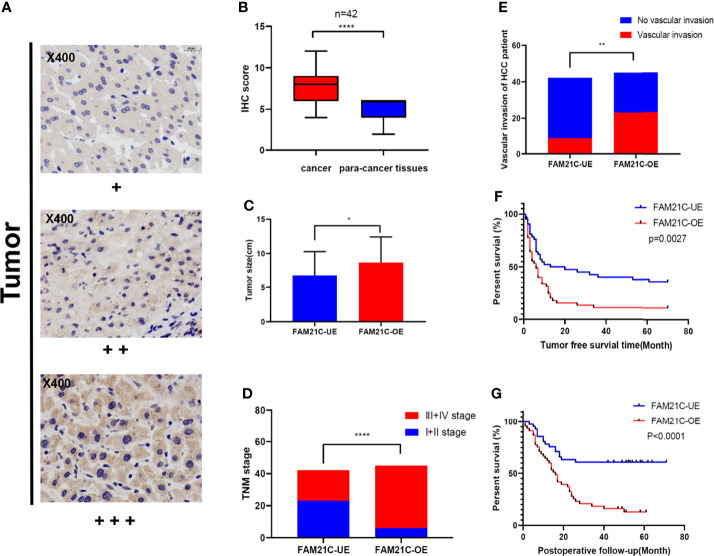
Up-regulated expression of FAM21C promotes the malignant progression of HCC in patients. **(A)** Immunohistochemical staining was performed in 129 HCC tissue. The stain intensity was classified as weak (+), moderate (++), or strong intensity (+++). Representative images are presented. FAM21C expression was scored according to intensity and area as described in the Materials and Methods. **(B)** The expression of FAM21C levels in 42 paired HCC tissue analyzed by paired Student’s *t* test. **(C)** The mean tumor size in HCC patient of the FAM21C low-expression and high-expression group; red indicates the overexpression and blue indicates the low-expression. **(D)** Tumor stage in HCC patients of the FAM21C overexpression and low-expression groups; red indicates the III+IV stage, and blue indicates I+II stage. **(E)** Vascular invasion in HCC patients of the FAM21C overexpression and low-expression groups; red indicates the occurrence of vascular invasion, and blue indicates no vascular invasion. **(F, G)** The Kaplan–Meier analysis of DFS and OS between FAM21C low-expression group and overexpression group. *P < 0.05, **P < 0.01, ****P < 0.0001.

To verify the prediction results of the bioinformatic analysis, a tissue microarray using primary HCC tissue samples was prepared. The tissue microarray contained 87 clinical tumor tissue samples collected from patients with HCC, 42 of which had paraneoplastic paired tissues. Seventeen (19.5%) of the 87 patients with HCC had TNM stage I, 12 (13.8%) had stage II, 44 (50.6%) had stage III, and 14 (16.1%) had stage IV. HCC was classified according to the degree of differentiation into a highly differentiated group: 8.0% (n=7), a moderately differentiated group: 75.9% (n=66), and a poorly differentiated group: 16.1% (n=14). Approximately 6.9% (n=6) of patients presented lymph node metastases, 36.8% (n=32) had vascular invasion, 77.0% (n=67) had recurrence within 5 years after surgery, 62.1% (n=54) patients died from cancer-related deaths within 5 years after surgery (**Table 1**). Subsequently, we performed immunohistochemical staining of FAM21C on HCC tissue microarrays, and the results showed that the tissues were classified as weakly positive (+), positive (++), and strongly positive (+++) according to the depth of staining (**Figure 2A**). Then we scored the 87 HCC tissues in the low expression group (n= 42) and the high expression group (n= 45) based on the score of the staining depth and the percentage of stained area, using the median as the cut-off value. Among the 42 pairs of cancer and para-cancer tissues, the expression of FAM21C was significantly higher in HCC tissues than in para-cancerous tissues (8.05 ± 2.34 *vs*. 4.86 ± 1.32, *P*<0.0001) (**Figure 2B**). Subsequently, we statistically analyzed pathological parameters such as tumor size, TNM stage, and vascular invasion, and postoperative follow-up data of HCC patients in the FAM21C low-expression and high-expression groups (**Supplementary Table 1**), and the results suggested that the tumor diameter of HCC in the FAM21C high-expression group was significantly greater than in the low-expression group (8.7 ± 0.6 cm *vs.* 6.8± 0.5 cm, *P*=0.0192) (**Figure 2C**). Thirty-nine patients in the FAM21C high-expression group were in stage III–IV, and the number was significantly higher than that of the 19 patients in the low-expression group (86.7% *vs.* 45.2%, *P*<0.001) (**Figure 2D**). Twenty-three patients in the FAM21C high-expression group presented microvascular invasion, which was significantly more pronounced than that 9 cases in the FAM21C low-expression group (51.1% *vs*. 21.4%, *P*=0.0071) (**Figure 2E**). Finally, we performed a statistical analysis of the prognosis of patients in the FAM21C high-expression and FAM21C low-expression groups, and the results showed that the OS and DFS rates were significantly lower in the FAM21C high-expression group than in the FAM21C low-expression group (**Figures 2F, G**). These results suggest that FAM21C is highly expressed in patients with HCC and can promote the malignant progression of HCC.

**Table 1:** Clinicopathologic Parameters of Patients.

Pathologic Variable	No. of Patients	Pathologic Variable	No. of Patients
TNM stage	87	Vascular invasion	87
Stage I	17 (19.5%)	Yes	32 (36.8%)
Stage II	12 (13.8%)	No	55 (63.2%)
Stage III	44 (50.6%)	Postoperative recurrence	87
Stage IV	14 (16.1%)	Yes	67 (77.0%)
HCC differentiation	87	No	20 (23.0%)
PD	14 (16.1%)	Cancer related death	87
MD	66 (75.9%)	Death	54 (62.1%)
WD	7 (8.0%)	Survival	33 (37.9%)
Lymph node metastasis	87		
Yes	6 (6.9%)		
No	81 (93.1%)		

### FAM21C Promotes HCC Cell Invasion and Migration *In Vitro*

**Figure 3:**
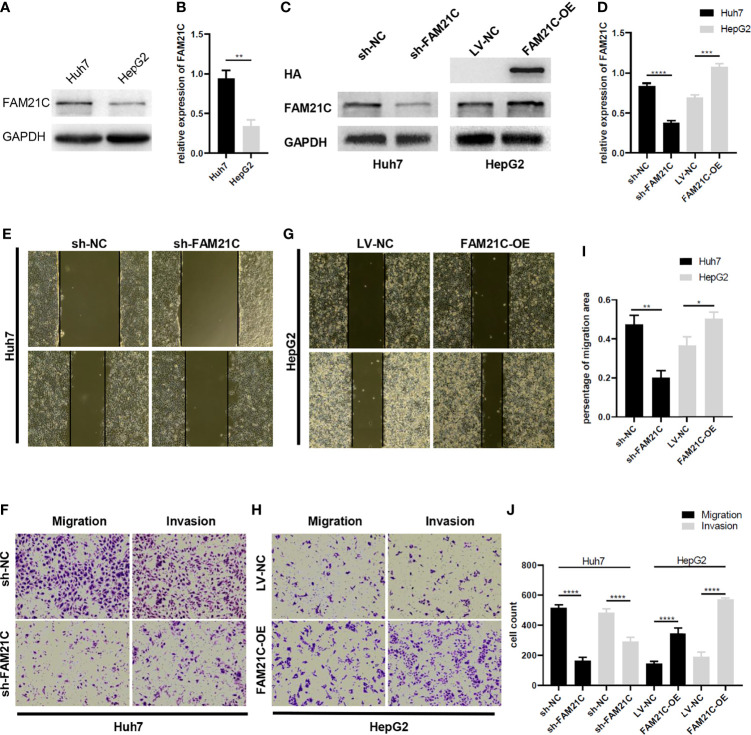
FAM21C increases HCC cell invasion and migration *in vitro*.**(A)** The wild-type FAM21C content of Huh7 and HepG2 cells was detected by western blot. **(B)** Histograms show the relative expression levels of FAM21C. **(C)** Western blotting was used to detect the protein levels of FAM21C in Huh7 and HepG2 after infected with sh-FAM21C and FAM21C-OE lentivirus compared with the respective negative control. HA indicated that the overexpression of HA-FAM21C fusion protein was effective. **(D)** Histograms show the relative expression levels of proteins. **(E–H)** Wound healing, Transwell and invasion assay were used to detect the migration and invasion potential of Huh7 and HepG2 cells after transfected with knockdown or overexpression lentivirus respectively. The invasion and migration ability of Huh7 was decreased after FAM21C knockdown; the invasion and migration ability of HepG2 was increased after FAM21C overexpression. Scale bar: 200×. **(I, J)** Histograms show the percentage of migration area and cell count after the FAM21C expression was modulated. Data are represented as the mean ± SD, n=3. *P < 0.05, **P < 0.01, ***P < 0.001, ****P < 0.0001.

The WASH complex is an important member of the WASP family that plays an important role in mediating the dynamic remodeling of the cytoskeleton. FAM21C is a key subunit of WASH; thus, we hypothesized that it might be associated with cytoskeleton-related invasion and metastasis. To investigate the role of FAM21C in HCC, we first tested the expression of FAM21C in high-invasive cells Huh7 and low-invasive cells HepG2. Our results revealed that the protein level of FAM21C in Huh7 cells was higher than in HepG2 (**Figures 3A, B**). Then we stably knocked down and overexpressed FAM21C in Huh7 and HepG2 cells respectively, while adding the HA tag to the overexpressing lentivirus to construct the HA-FAM21C fusion protein (**Figures 3C, D**). Transwell and Wound healing assays showed that the migration ability of Huh7 cells with stably knocked down FAM21C expression was significantly reduced; and the invasion ability was also significantly reduced compared to the negative control in Matrigel-precoated chambers (**Figures 3E, F, I, J**). In contrast, in HepG2 overexpressing FAM21C, migration ability was enhanced with increased expression of FAM21C and invasion ability was also significantly increased (**Figures 3G–J**). Reciprocally, we overexpressed FAM21C in Huh7 cells and knocked it down in HepG2, finding that the invasive and migratory ability of Huh7 and HepG2 was dramatically increased and significantly decreased respectively (**Supplementary Figure 2**). These results suggested that FAM21C can promote the invasive and migratory ability of HCC cells *in vitro*.

### FAM21C Is Involved in Regulating Actin Filament Cytoskeleton Remodeling Through Binding to CAPZA1

**Figure 4:**
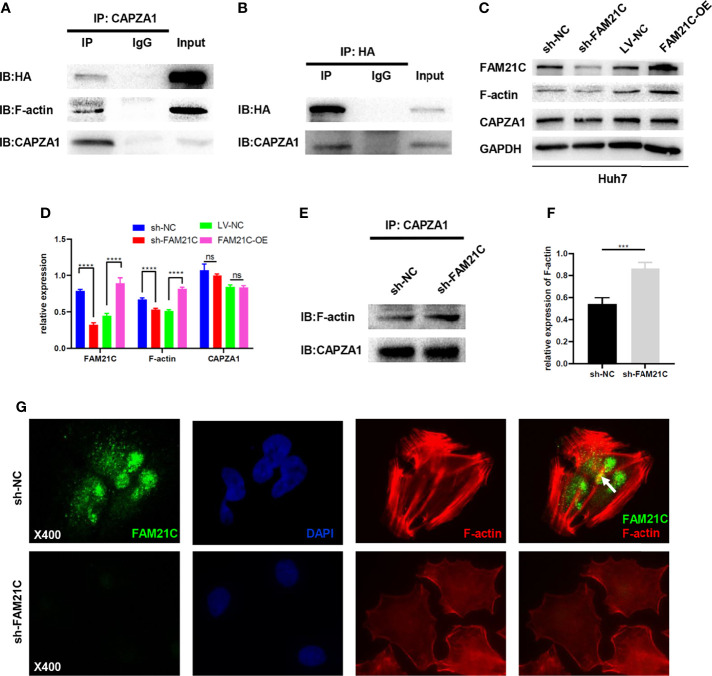
FAM21C bind to CAPZA1 and inhibit the capping ability of CAPZA1 to promote cytoskeleton remodeling in HCC cells. **(A, B)** Co-Immunoprecipitation(Co-IP) was performed to validate protein interactions. The results showed that HA-FAM21C and CAPZA1 can pull each other down. Moreover, Co-IP also showed that CAPZA1 can interact with F-actin. **(C)** Western blotting was used to detect the expression of FAM21C, F-actin, and CAPZA1. GAPDH was used as the loading control. **(D)** Histograms show the relative expression. **(E)** Quantitative Co-Immunoprecipitation showed that the binding level of F-actin to CAPZA1 was increased after FAM21C knockdown. CAPZA1 level was considered loading control. **(F)** After Quantification by ImageJ software, the histograms showed the relative level of F-actin. **(G)** Immunofluorescence assay on Huh7 cells showed that with the decreased of FAM21C, actin cytoskeleton scattered arrangement, and lack of the colocalization of FAM21C and F-actin. (The arrowhead shows the colocalization). Nuclei were stained with DAPI. Scale bar = 400×. Data are represented as the mean ± SD, n=3. ***P < 0.001, ****P < 0.0001; ns for no significance.

A previous study by our group found that low expression of CAPZA1 induced the remodeling of the actin filament cytoskeleton in HCC cells, driving EMT and thus promoting invasion and migration of HCC cells (11). A quantitative/TMT IP-MS analysis of KIAA0196 indicated that FAM21C could interact with CAPZA1 (24). What’s more, The bioinformatics analysis also showed that the two can be combined (**Supplementary Figure 3**), but the effects of its combination on HCC cells are unclear. Thus, we used immunoprecipitation assays to first verify whether FAM21C could interact with CAPZA1 in HCC cells by constructing an FAM21C-HA fusion protein using a HA tag, and interfering FAM21C expression with shRNA. Magnetic beads encapsulated with the CAPZA1 antibody were used in pull-down HA tag from the total protein lysate. The results showed that the two could bind to each other in Huh7 overexpressing the FAM21C-HA fusion protein, indirectly verifying that FAM21C could interact with CAPZA1 (**Figures 4A, B**). Consistent with our previous study, immunoprecipitation assays confirmed that CAPZA1 could bind to F-actin (**Figure 4A**). The effect of FAM21C bind to CAPZA1 on the F-actin cytoskeleton was then explored. Using western blotting assays, Huh7 cells with knockdown of FAM21C, CAPZA1 expression did not change, but the protein level of F-actin was decreased; in contrast, in Huh7 cells overexpressing FAM21C-HA, CAPZA1 expression was also unchanged, but the protein level of F-actin was increased (**Figures 4C, D**). The results suggest that FAM21C is not significantly related to the expression of CAPZA1, but may be involved in regulating the level of F-actin. A subsequent search of the GEPIA database revealed no significant correlation between FAM21C mRNA levels and F-actin (**Supplementary Figure 4**). This suggested to us that FAM21C may influence the intracellular level of F-actin by promoting its remodeling. Next, we performed quantitative Co-IP experiments by collecting the same number of Huh7 cells in the FAM21C knockdown and control groups, extracting the total protein and then normalizing the protein concentration, and verifying the amount of F-actin bounding by enriching CAPZA1. The results showed that after FAM21C was knocked down, the amount of F-actin bound to CAPZA1 increased significantly in contrast to the decrease in total F-actin content (**Figures 4E, F**). Meanwhile, immunofluorescence assays revealed that the amount of FAM21C was significantly reduced in the knockdown group compared to the control group, and the actin cytoskeleton was scattered, while there was a lack of colocalization of FAM21C with F-actin (**Figure 4G**). These results suggested that FAM21C can promote F-actin polymerization and thus regulate actin cytoskeleton remodeling by interacting with CAPZA1 and inhibiting the capping ability of CAPZA1 in HCC cells.

### FAM21C Binds to CAPZA1 Mainly Through the CPI Domain and Inhibits the CAPZA1 Capping Function, Thus Promoting the Invasion and Migration of HCC Cells

**Figure 5:**
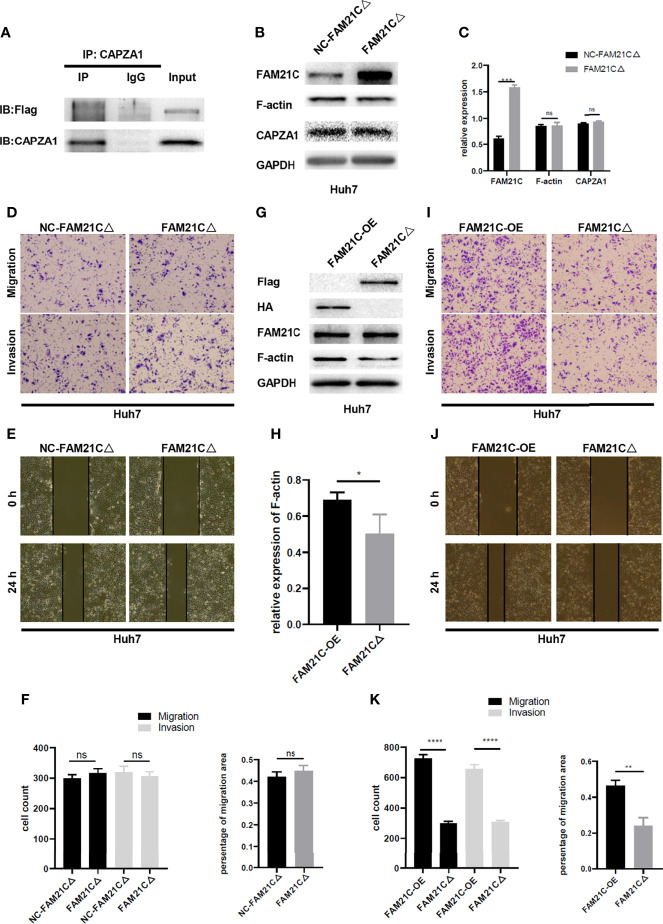
FAM21C CPI mutation failed to promote the HCC cells invasion and migration. **(A)** Co-IP was used to validate the interaction between FAM21C mutation and CAPZA1. The results showed that CPI mutation interfered with binding to CAPZA1. **(B)** Western blotting was performed to detect the expression level of FAM21C, CAPZA1 and F-actin following transfected with mutation lentivirus in Huh7 cells. **(C)** Histograms showed the relative expression level. **(D, E)** Transwell, Wound healing, and invasion assays were performed to validate the migration and invasion potential of CPI mutation Huh7 cells. **(F)** Histograms showed the results were of no significant. **(G)** The protein of Flag, HA, FAM21C, F-actin, GAPDH were analyzed by western blotting. **(H)** Histograms showed the relative expression levels of F-actin. **(I, J)** Transwell, Wound healing, and invasion assays were performed to validate the migration and invasion potential of FAM21C-OE-Huh7 and FAM21CΔ-Huh7 cells. **(K)** The statistical graph indicates the cell count and percentage of the migration area. Data are represented as the mean ± SD, n=3. *P < 0.05, **P < 0.01, ***P < 0.001, ****P < 0.0001; ns for no significance.

Protein molecules containing the CPI domain can bind to CAPZ; leucine, arginine, proline, which are 3 highly conserved amino acids exist in the CPI domain (25); thus, we performed a targeted mutation of these three conserved amino acids to alanine in the CPI domain based on the amino acid sequence of FAM21C (26)(**Supplementary Figure 1**), to further verify whether FAM21C promotes the invasion and migration of HCC cells by binding to CAPZA1. We constructed a fusion protein (FAM21CΔ-Flag) with the Flag tag protein and mutant FAM21C. An immunoprecipitation was performed to verify whether the Flag-tagged mutant FAM21C could bind to CAPZA1. The results showed that the two did not bind (**Figure 5A**). Subsequently, immunoblotting assay was performed to detect transfection efficiency and the effect of mutant FAM21C on actin cytoskeleton. The results showed that the content of FAM21C was significantly higher in the treated group compared to the control group, but the content of CAPZA1 and F-actin were not significantly different (**Figures 5B, C**), suggesting that the mutant FAM21C failed to affect the actin cytoskeleton of Huh7 cells. Then, *in vitro* functional assays did not show any significant differences in the migration and invasion ability of Huh7 cells transfected with mutant FAM21C compared with the control group (**Figures 5D–F**). To go a step further, we compared the biological role of wild-type FAM21C and mutant FAM21C *via* FAM21C-OE-Huh7 and FAM21CΔ-Huh7 cells. The western blotting assay showed that the F-actin level was significantly elevated in FAM21C-OE-Huh7 cells compared with FAM21CΔ-Huh7 cells (**Figures 5G, H**). Consistent with these findings, the invasive and migratory ability of FAM21C-OE-Huh7 cells was enhanced compared to FAM21CΔ-Huh7 cells (**Figures 5I–K**). These data demonstrated that the binding of FAM21C to CAPZA1 was inhibited by the mutation of the CPI domain, resulting in an inability of FAM21C to regulate actin cytoskeleton through CAPZA1, which failed to influence the invasion and migration ability of HCC cells. In conclusion, FAM21C exerts its procarcinogenic effects by binding to the CAPZA1 through the CPI domain, which in turn induces remodeling of the F-actin cytoskeleton, thus promoting HCC cells invasion and migration.

### FAM21C Promotes Invasion and Metastasis of HCC in Nude Mice

**Figure 6:**
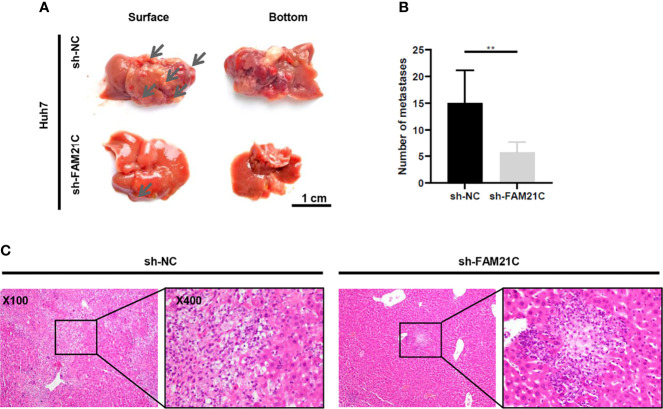
FAM21C could promote HCC invasion and metastasis *in vivo.* **(A)**The metastatic tumor foci were widely distributed on the liver surface of the negative control group. The few metastatic lesions were localized predominantly on the liver lobe of the sh-FAM21C-expressing Huh7 group. Scale bar:1cm. **(B)** Histograms show the number of the metastatic tumor foci. Data are represented as the mean ± SD, n=6. **P < 0.01. **(C)** Haematoxylin and eosin staining was performed on xenograft liver sections. Scale bar = 100× and 400×.

We have demonstrated that FAM21C promotes HCC cell invasion and migration *in vitro*. To investigate the role of FAM21C *in vivo*, sh-FAM21C-expressing Huh7 and its negative control cells were injected into the left lobe of the nude mice liver (n=6). Six weeks later, all nude mice were deeply anesthetized and euthanized, and the metastatic foci on the surface of the nude mice liver was observed. The result showed that few metastatic foci were present on the liver surface of nude mice in the sh-FAM21C-expressing Huh7 group; in contrast, in the control group, more metastatic foci were diffused on the liver surface, along with the formation of localized masses (**Figures 6A, B**). Subsequently, The liver sections were confirmed as tumors tissue by hematoxylin and eosin staining. The result showed that in the control group the metastasis foci were more widely distributed in the liver tissue; Conversely, the distant metastatic lesions of the sh-FAM21C-expressing Huh7 group displayed a restricted distribution (**Figure 6C**) (**Supplementary Figure 5**). This result suggested that FAM21C could promote the invasive and migratory ability of HCC cells *in vivo.*

## Discussion

In the present study, FAM21C expression was up-regulated in HCC tissues and its high expression was significantly associated with malignant progression of HCC. Meanwhile, we confirmed that FAM21C could promote the invasion and metastasis of HCC *in vitro* and *in vivo*. Additionally, we found and confirmed that FAM21C could interact with CAPZA1 through its CPI domain in HCC cells and FAM21C inhibits the capping ability of CAPZA1, thus inducing the remodeling of the actin filament cytoskeleton, which in turn promoted HCC invasion and metastasis.

During tumor development, tumors achieve distant settlement by encroaching on surrounding tissues to break the tumor barrier (27). Dynamic remodeling of the actin filament cytoskeleton and its associated regulators play an unquestionable role in tumor spreading (28, 29). Thus it is important to investigate the mechanism of actin filament cytoskeleton remodeling during invasion and migration of HCC cells to improve the patient’s prognosis. Based on the findings of a previous study, we investigated the mechanism by which FAM21C induces the remodeling of the F-actin cytoskeleton by regulating CAPZA1 to promote HCC invasion and metastasis. Consistently, the dynamic remodeling of F-actin includes nucleation, polymerization, depolymerization, and side branch formation (30). The WASP family molecules are important regulators of F-actin remodeling, and their VCA domains function to promote actin filament nucleation and lengthening and can play important roles in different cellular substructures (31). For example, N-WASP, an important member of WASP, is highly expressed in HCC, and its elevated expression predicts a poor prognosis in HCC patients, and the potential mechanism maybe to promote prolongation of actin filament side branch polymerization by activating the Arp2/3 complex, which drives the formation of a bulge in the cell membrane and thus enhances cell migration (32, 33). WASH, a newly identified member of the WASP family, can regulate actin formation on the surface of the endosomal membrane to form a reticular scaffold, thus participating in regulation of transferrin recycling (34). Furthermore, in esophageal cancer, WASH overexpression improves the characteristics of tumor stem cells and is associated with a poor prognosis (35). The above results suggest that the WASP family may play a role as a pro-oncogene in tumors, in which the WASH complex may promote tumor cell development by regulating actin filament cytoskeleton remodeling, but the exact mechanism is not yet clear. FAM21C is an important subunit of the WASH complex, and studies have shown that FAM21 knockdown reduces the protein level of WASH, but WASH knockdown does not alter FAM21 expression (36). In this study, we investigated the potential mechanism of FAM21C, a key subunit of the WASH complex, to promote the invasion and metastasis of HCC through regulation of the remodeling of the F-actin cytoskeleton, furthering the understanding of the mechanism of the WASP family proteins to promote the invasion and metastasis of HCC.

CAPZA1 is an important molecule that regulates the remodeling of F-actin, which mainly regulates the prolongation of F-actin by binding at the barbed end and preventing the polymerization of G-actin. In our previous study, we found that CAPZA1 had low expression in HCC tissues and its low expression could promote the malignant progression of HCC by regulating the remodeling of the F-actin cytoskeleton to promote EMT in HCC cells (11). Furthermore, in our recent study, we found that in HCC cells, PIP2 can bind to CAPZA1, and the combination of the two led to disengaging CAPZA1 from the barbed end of F-actin, which in turn promoted the prolongation of F-actin and drove the morphogenesis of HCC cells (20). In this study, we found that FAM21C was highly expressed in HCC tissues by bioinformatics analysis, and its high expression predicted a poor prognosis for HCC patients; Meanwhile, we further verified that FAM21C was highly expressed in HCC tissues using TMAs, and its high expression could promote malignant progression of HCC, and this result was also confirmed *in vitro* and *in vivo*. Subsequently, we explored the molecular mechanisms involved in FAM21C that promotes HCC cell invasion and migration. We found that FAM21C could bind to CAPZA1, and its binding could inhibit the capping ability of CAPZA1 and thus promote the prolongation of F-actin. After mutating the binding site, FAM21C failed to regulate actin cytoskeleton *via* CAPZA1 capping ability. Therefore, we concluded that FAM21C plays an important role in the invasion and metastasis of HCC by inhibiting capping ability by binding to CAPZA1, leaving the barbed end of F-actin in an open state and polymerizing in the positive direction, ultimately promoting dynamic remodeling of the actin cytoskeleton.

It has been reported that FAM21C can attach the WASH complex to endosomal membranes and is an essential molecule for retromer-mediated WASH-dependent sorting of cargo transport (36). In addition, WASH-mediated transport of endosomal cargo such as β1 integrin and MT1-MMP is important for tumor cell invasion and migration (37, 38). Furthermore, the presence of numerous retromer-binding sites in FAM21C allows the cargo, retromer, and WASH complexes to constitute a fluid sorting platform linked to the actin cytoskeleton (39). These findings suggested that FAM21C, a key subunit of the WASH complex, is not only an essential molecule to maintain the stability and function of the WASH complex, but also plays an important role in actin cytoskeleton-dependent endosomal vesicle transport. In particular, the regulation of the specific endosomal cargo transport can have a significant impact on the behavior of tumor cells such as invasion and migration. Moreover, it was also reported that DKO cells transfected with the FAM21 siRNAs, along with the loss of actin foci (comets) on endosomes (40). In this study, the co-localization of FAM21C with the actin cytoskeleton was detected using an immunofluorescence assay, suggesting a potential association between the subcellular structure in which FAM21C is located and the actin cytoskeleton. Therefore, we hypothesize that during HCC cell invasion and migration, FAM21C in endosomal membranes remodels the F-actin cytoskeleton through regulation of CAPZA1, an event that promotes endosomal membrane skeleton formation and prepares the structure for endosomal vesicle transport and sorting. The above results suggest that FAM21C-regulated remodeling of the F-actin cytoskeleton is closely related to endosome-dependent cargo transport. Unfortunately, we did not investigate the related mechanisms in depth in this study, although we plan to design a specific study in the future.

In summary, we conclude that the high expression of FAM21C in HCC tissues can promote the malignant progression of HCC, and its mechanism involves the inhibition of CAPZA1 capping function by FAM21C which binds to CAPZA1, leaving the F-actin barbed-end in an open state, which in turn induces the remodeling of the F-actin cytoskeleton, thus promoting the invasion and migration of HCC cells. The remodeling of the F-actin cytoskeleton regulated by FAM21C through CAPZA1 may be closely related to endosome-dependent cargo transport, which deserves further in-depth study and its potential to become a new target for the treatment of HCC.

## Data Availability Statement

The original contributions presented in the study are included in the article/**Supplementary Material**. Further inquiries can be directed to the corresponding author.

## Ethics Statement

The studies involving human participants were reviewed and approved by the Institutional Research Ethics Committee of Southwest Hospital. The patients/participants provided their written informed consent to participate in this study. The animal study was reviewed and approved by the Laboratory Animal Welfare and Ethics Committee of the Third Military Medical University (Army Medical University), Chongqing, China. Written informed consent was obtained from the individual(s) for the publication of any potentially identifiable images or data included in this article.

## Author Contributions

YL designed and performed experiments, analyzed data and wrote the paper. DH analyzed the data and revised the manuscript. BW, BZ, JL, and JS performed a part of experiments. SZ initiated the study, provided the financial support and supervised laboratorial processes. All the authors approved the final manuscript.

## Funding

This research was funded by the National Natural Science Foundation of China, grant number 81972303.

## Conflict of Interest

The authors declare that the research was conducted in the absence of any commercial or financial relationships that could be construed as a potential conflict of interest.

## Publisher’s Note

All claims expressed in this article are solely those of the authors and do not necessarily represent those of their affiliated organizations, or those of the publisher, the editors and the reviewers. Any product that may be evaluated in this article, or claim that may be made by its manufacturer, is not guaranteed or endorsed by the publisher.

## Abbreviations

CAPZA1, capping actin protein of muscle Z-line alpha subunit 1; Co-IP, Co-immunoprecipitatio; F-actin, actin filament; HCC, hepatocellular carcinoma; IB, Immunoblot; IP, Immunoprecipitation; MD, moderately differentiated; PD, poorly differentiated; WD, well differentiated.
