# Supplementary material for: FAM21C Promotes Hepatocellular Carcinoma Invasion and Metastasis by Driving Actin Cytoskeleton Remodeling via Inhibiting Capping Ability of CAPZA1
Source: Front Oncol. 2022 Jan 13;11:809195. doi: 10.3389/fonc.2021.809195 (PMC8793146; doi:10.3389/fonc.2021.809195)
Supplement: Supplementary file 2 [file DataSheet_2.docx]

Supplementary Material

# Supplementary Figures and Tables

## Supplementary Figures

| Protein | Amino Acid Sequence (L1003A/R1010A/P1019A) |
| --- | --- |
| FAM21C (WT) | LHSANKSR VKMRGKRRP |
| FAM21C△（CPI*） | AHSANKSA VKMRGKRRA |

**Supplementary Figure 1.** Schematic diagram of mutation site of FAM21C. Amino Acid Sequence of FAM21C.


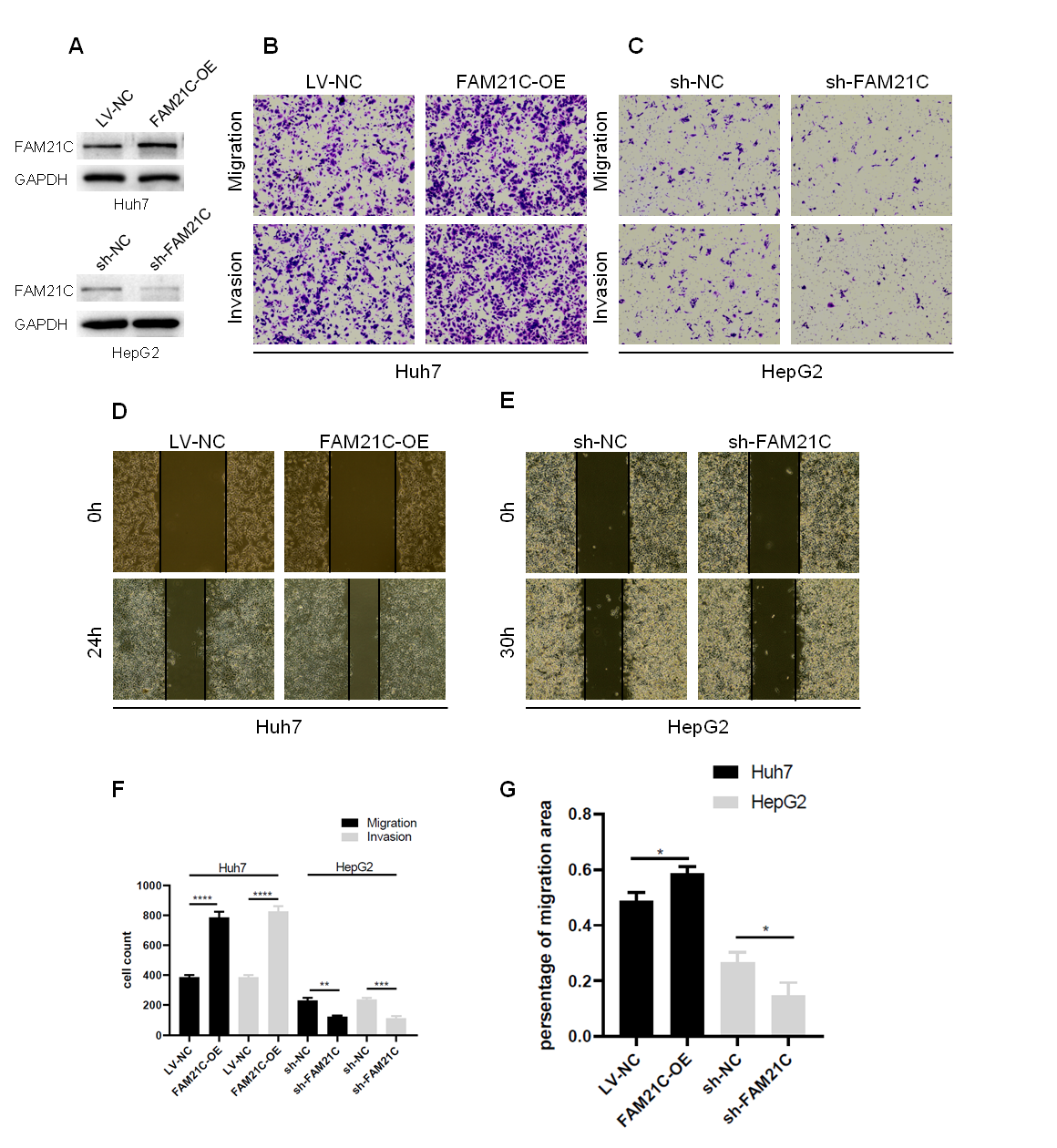


**Supplementary Figure 2.** FAM21C promotes HCC cell invasion and migration *in vitro*.**(A)** Western blotting was used to detect the protein levels of FAM21C in Huh7 and HepG2 after infected with FAM21C-OE and sh-FAM21C lentivirus compared with the respective negative control. **(B-E)** Wound healing, Transwell, and Invasion assay were used to detect the migration and invasion potential of Huh7 and HepG2 cells after transfected with overexpression or knockdown lentivirus respectively. The invasion and migration ability of Huh7 was increased after FAM21C overexpression; the invasion and migration ability of HepG2 was decreased after FAM21C knockdown. Scale bar: 200×. **(F,G)** Histograms show the percentage of migration area and cell count after the FAM21C expression was modulated. Data are represented as the mean ± SD, n=3.


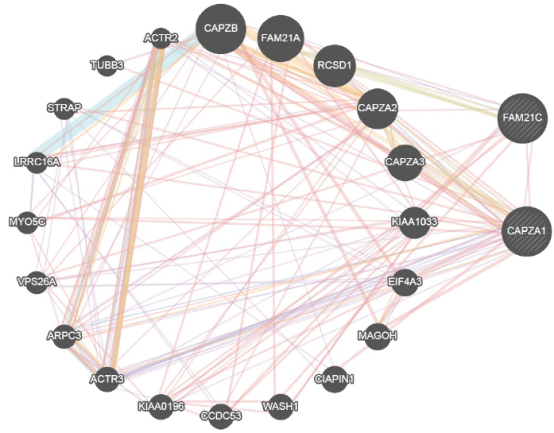

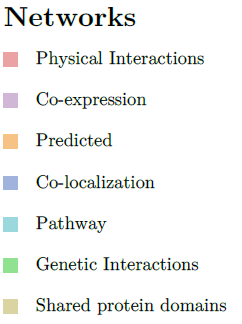


**Supplementary Figure 3.** Bioinformatics analysis showed that FAM21C could bind to CAPZA1 by GeneMENIA database.


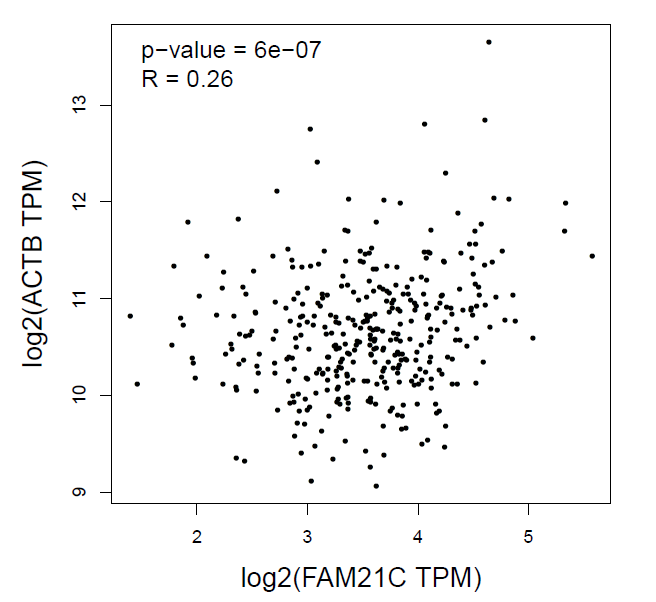


**Supplementary Figure 4.** Bioinformatics analysis showed that the mRNA level of FAM21C and F-actin do not exist significant correlation.


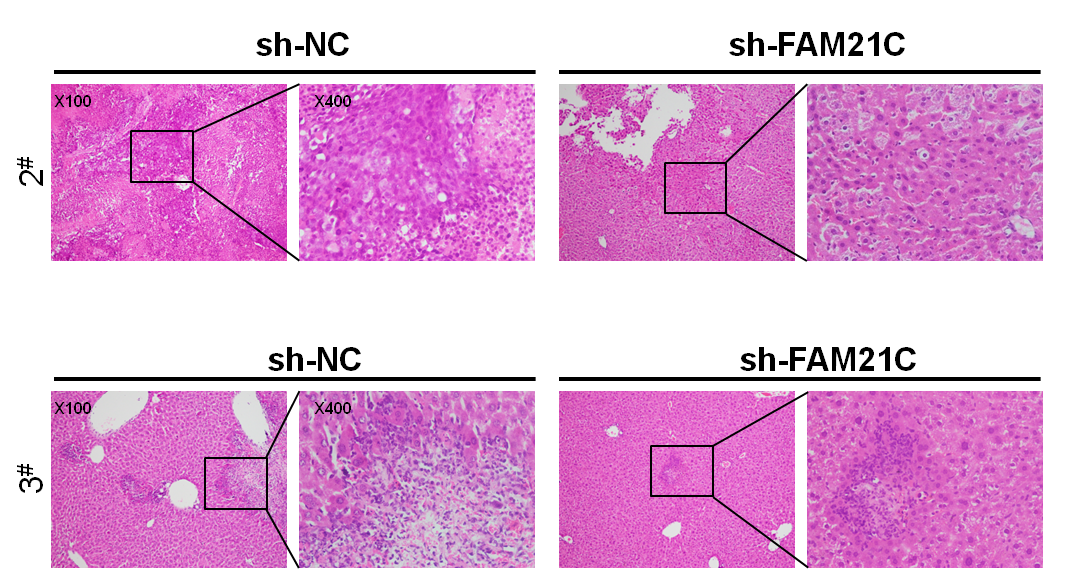


**Supplementary Figure 5.** Haematoxylin and eosin staining was performed on xenograft liver sections. Scale bar = 100× and 400×.

## Supplementary Tables

|  | The level of FAM21C expression | | *P*-value |
| --- | --- | --- | --- |
|  | Underexpression  2,3,4,6,8 | Overexpression  9，12 |  |
| Age | 49.0±1.5 | 46±1.5 | 0.1822 |
| Mean tumor size（cm） | 6.8±0.5 | 8.7±0.6 | **0.0192** |
| TNM stage |  |  | **<0.001** |
| Stage I-II | 23/42（54.8%） | 6/45（13.3%） |  |
| Stage III-IV | 19/42（45.2%） | 39/45（86.7%） |  |
| HCC differentiation |  |  | 0.0903 |
| PD | 5/42（11.9%） | 9/45（20.0%） |  |
| MD | 31/42（73.8%） | 34/45（75.6%） |  |
| WD | 6/42（14.3%） | 1/45（2.2%） |  |
| Lymph node metastasis | 2/42（4.8%） | 4/45（8.9%） | 0.6774 |
| Vascular invasion | 9/42（21.4%） | 23/45（51.1%） | **0.0071** |
| Postoperative recurrence | 27/42（64.3%） | 40/45（88.9%） | **0.0027** |
| Cancer related death | 16/42（38.1%） | 38/45（84.4%） | **<0.0001** |

**Supplementary Table 1** The relationship between clinicopathological features and FAM21C.
